# Effect of dietary fat type on anxiety-like and depression-like behavior in mice

**DOI:** 10.1186/2193-1801-2-165

**Published:** 2013-04-16

**Authors:** Wataru Mizunoya, Koichiro Ohnuki, Kento Baba, Hideo Miyahara, Naomi Shimizu, Kuniko Tabata, Takako Kino, Yusuke Sato, Ryuichi Tatsumi, Yoshihide Ikeuchi

**Affiliations:** 1 Department of Bioresource Sciences, Graduate School of Agriculture, Kyushu University, 6-10-1 Hakozaki, Fukuoka, Higashi-ku, 812-8581 Japan; 2 Faculty of Food and Nutrition, Kyushu Nutrition Welfare University, 5-1-1 Shimoitouzu, Kitakyushu, Kokurakita-ku, 803-8511 Japan

**Keywords:** Fish oil, Lard, Soybean oil, Anxiety, Depression

## Abstract

Dietary fat plays an important role in higher brain functions. We aimed to assess the short and long term intake of three different types of dietary fat (soybean oil, lard, and fish oil) on anxiety-like and depression-like behavior in mice. For the short term intake assessment, a behavioral test battery for anxiety and depression was carried out for a 3-day feeding period. For the long term intake assessment, a behavioral test battery began after the 4-week feeding period. During the short term intake, the time spent in the open arms of the elevated plus-maze was the longest in the fish oil fed group, followed by the soybean oil and lard-fed groups. The elevated plus-maze is a common animal model to assess anxiety, in which an increased time spent in the open arms indicates an anxiolytic effect. The difference between the fish oil-fed group and lard-fed group was statistically significant (p < 0.01), but there was no significant difference between the soybean oil-fed group and the other two groups. Similar results were observed after a 4-week feeding period. On the other hand, there was no significant difference among the three groups in behavior tests to evaluate depression. Thus, the dietary fat types appeared to influence anxiety but not depression in mice, both in short term (3 days) and long term (4 weeks) feeding.

**Electronic supplementary material:**

The online version of this article (doi:10.1186/2193-1801-2-165) contains supplementary material, which is available to authorized users.

## Background

There are many kinds of fatty acids that are grouped into three main classes: saturated, monounsaturated, and polyunsaturated fatty acids (PUFA). Neither n-6 PUFA (e.g. linoleic acid) nor n-3 PUFA (e.g. alpha-linoleic acid) can be synthesized by mammals. In food, the proportions of n-6/n-3 PUFA are variable. In the phospholipids of animal cell membranes, the n-6/n-3 PUFA ratio is changed by food consumption. This change also influences the cell membrane’s fluidity in the central nervous system (Hashimoto et al. 2001), and shifts of the fatty acid composition in cell membranes alter the activity of receptors and proteins (Bourre et al. 1989; Foot et al. 1983; Taffet et al. 1993). It is suggested that these changes may affect the higher brain functions. Interestingly, it was revealed that some neurotransmitters, such as endocannabinoids, are synthesized from fatty acids (Devane et al. 1992), suggesting that fatty acid metabolites derived from dietary fat can affect the central nervous system.

To date, various physiological functions of n-3 PUFA are studied. Fish oil is a dietary source that is high in docosahexaenoic acid (DHA) and eicosapentaenoic acid (EPA); types of n-3 PUFA that are absent from most other oils. Many studies have shown that n-3 PUFA are related to animal learning performance (Bourre et al. 1989; Gamoh et al. 2001; Gamoh et al. 1999; Nakashima et al. 1993). At present, some researchers are examining the effect of n-3 PUFA deficiency on anxiety by using the elevated plus-maze test. These results are controversial, as in the literature, n-3 PUFA deficiency increases (Carrie et al. 2000; Takeuchi et al. 2003), decreases (Frances et al. 1995; Nakashima et al. 1993), and does not change (Belzung et al. 1998; Fedorova et al. 2007) the time spent in the open arms in the elevated plus-maze test. Lafourcade et al. showed that lifelong n-3 PUFA deficiency exhibited more anxiogenic behavior in the open field test than n-3 PUFA diet mice and involvement in endocannabinoid-mediated neuronal activity (Lafourcade et al. 2011). Note that all of these studies were based on a dietary deficiency of n-3 PUFA, rather than an addition of n-3 PUFA in the diet. Furthermore, the effect of fish oil, rather than n-3 PUFA intake, on anxiety-like behavior in mice has yet to be reported on.

There is a suggested relationship between n-3 PUFA and depressive symptoms. There is a negative correlation between the incidence of depression and the amount of fish intake (Hibbeln 1998) and the plasma phospholipid n-6/n-3 ratio (Kiecolt-Glaser et al. 2007). It is further reported that supplemental n-3 PUFA in the diet causes significant improvement in depressive symptoms in humans (Peet and Horrobin 2002). A recent study in humans showed that diets with EPA ≥ 60% of total DHA + EPA had an effect against depression in a meta-analysis of 15 clinical studies on depression related to PUFA in diet (Sublette et al. 2011).

Dietary fat derived from plants, animals, and fish has different and particular fatty acid profiles. Our purpose is to assess the short and long term intake of three different types of dietary fat (soybean oil, lard, and fish oil) on anxiety-like and depression-like behavior in mice. Soybean oil is often treated as a control dietary oil in animal experiments, because it is adopted in AIN-93G or AIN-93M formulae diets (Reeves 1997). However, we did not define the control dietary oil in this experiment and compared these three dietary oils in parallel.

## Methods

### Animal

Five (for long term feeding) or 9 (for short term feeding) weeks old male C57BL/6J mice (CLEA Japan Inc.) were housed in groups of four in standard cages (33 × 23 × 12 cm) under controlled conditions of temperature (22 ± 2°C), relative humidity (50 ± 10%), and lighting (lights on from 8:00 to 20:00). They were given free access to water and a diet. They were housed under these conditions for at least 1 week preliminarily prior to the start of the experiments. During the preliminary period, all mice were supplied with a basal diet, CRF-1 (Charles River Laboratories Japan Inc.). All behavioral tests were carried out between 9:00 and 18:00 at 10–11 weeks of age. All animal experiments were conducted according to institutional guidelines and with the approval of Kyushu University Institutional Review Board.

### Experimental diets

Table 1 shows the composition of diets used in this experiment. These diets that contain 15% (w/w) soybean oil, lard, or fish oil, respectively, were prepared and made into pellets by Oriental Yeast. Co. Ltd, in which the energy ratio of fat was 31%. The fish oil used in the diet was supplied by Nippon Chemical Feed Co. Ltd.

**Table 1:** **The composition of experiment diets (g/kg)**

Ingredient	Soybean oil diet	Lard diet	Fish oil diet
α-Corn starch	387.202	387.202	387.202
Sucrose	120.514	120.514	120.514
Casein	241.029	241.029	241.029
Soybean oil	150.000	-	-
Lard	-	150.000	-
Fish oil	-	-	150.000
Cellulose powder	50.000	50.000	50.000
Mineral mix (AIN-93G-MX)	35.000	35.000	35.000
Vitamin mix (AIN-93-VX)	10.000	10.000	10.000
L-Cystine	3.615	3.615	3.615
Choline bitartrate	2.500	2.500	2.500
*tert-*Butylhydroquinone	0.140	0.140	0.140

### The fatty acid composition of dietary fat

The fatty acid composition of soybean oil, lard, and fish oil was analyzed by gas chromatography (GC-14A, Shimadzu Co. Ltd.) using an instrument equipped with a capillary column (0.25 mm × 30 m; HR-SS-10, Shinwa Chemical Industries Ltd.) and a hydrogen-flame ionization detector, according to the method described previously (Kawahara et al. 1997).

### Experimental scheme

In the short term feeding (3 days), behavioral tests were carried out on Day 1, 2, and 3 (n = 24/group). In the long term feeding (4 weeks), behavioral tests were carried out after the 4-week feeding period (n = 12/group). The animals were allowed free access to experimental diet and water during the whole experimental period. Pair-feeding was not conducted to avoid the effect of fasting on behavioral tests. The experimental diets were also given during the behavioral testing period continuously. We designed the behavioral battery tests according to a previous report (Arron et al. 2006; Miyakawa et al. 2003; Takao and Miyakawa 2006; Zeng et al. 2001) with a slight modification. The behavioral battery tests include the light–dark transition test, open field test, elevated plus-maze test and social interaction test to evaluate anxiety and Porsolt forced-swim test and tail-suspension test to evaluate depressive symptoms. The behavioral tests were done according to the following order: 1. light–dark transition test, 2. open field test, 3. elevated plus-maze test, 4. social interaction test, 5. Porsolt forced-swim test, and 6. tail-suspension test. This order was sorted based on the extent of presumed stress; i.e., earlier tests are less stressful. Only one type of behavioral test was done in any one day, to limit stress to the animals. In the short term feeding, different mice were used for the first 3 behavioral tests and the later 3 tests, because the feeding period was limited to 3 days. We also examined the antidepressive effect of dietary fat with depressive mice induced by reserpine (Sigma-Aldrich Co.), an antihypertensive drug that depletes neuronal storage granules of norepinephrine, serotonin, and dopamine in brain (Goodwin and Bunney 1971), by using different mice in the short term feeding experiment or mice finishing 6 behavioral battery tests in the long term feeding experiment. Reserpine was injected intraperitoneally (2 mg/kg) 42 and 18 h before the Porsolt forced-swim test was conducted and 18 h before the tail-suspension test was performed. All mice were experimentally naive; i.e. mice never repeated the same behavioral test a second time, except for the mice that were tested using the Porsolt forced-swim and tail-suspension tests with reserpine injection in the long term feeding study.

### Light–dark transition test

The apparatus used for the light–dark transition test consisted of a cage (21-L × 42-W × 25-H cm) divided equally into 2 by a partition containing a small opening. One chamber was made of white plastic and was brightly illuminated (500 lux), whereas the other chamber was black and dark. Mice were allowed to move freely between the 2 chambers for 10 min.

### Open field test

The open fields test was conducted with an open-field apparatus (50-L × 50-W × 30-H cm). The floor of the apparatus was compartmentalized into 5 × 5 = 25 square areas (10 cm square × 25) on the computer software, and those central 4 × 4 = 16 square areas were considered as the central part. Mice were put into the apparatus from the left-hand corner and were allowed to move freely for 10 min. The test was carried out under high-light (500 lux) conditions.

### Elevated plus-maze test

The elevated plus-maze consisted of an enclosure with 2 open arms (30-L × 5-W cm in size) and 2 enclosed arms of the same size with transparent walls 15 cm high. The arms were made of white acrylic plates radiating from a central platform (5 × 5 cm) to form a “plus” sign. The entire apparatus was elevated to a height of 55 cm above floor level. Each mouse was placed in the central platform facing one of the closed arms. The time spent on the open and closed arms and the central platform was recorded for 10 min. The light intensity was 220 lux in the open arms and 130 lux in the closed arms.

### Social interaction test

The social interaction test was similar to that described by Sankoorikal et al. (2006), with modifications appropriate for our facility and setting. The same arena of the Open field test was used, and 2 transparent plastic containers, shaped like a triangular prism (10-L × 10-W × 20-H cm, without a ceiling) and displaying several lower holes to allow mice to smell and touch one another, were installed in face-to-face corners of the arena. The area around the container into which the “stimulus mouse” would be introduced was arbitrarily designated the “social area,” and the area around the empty container at the opposite corner was designated the “nonsocial area” (12.5 cm^2^ each). The times spent by a subject mouse in the social area, nonsocial area, and other areas were measured for 10 min.

### Porsolt forced-swim test

The forced-swim test was performed essentially according to the procedures described by Porsolt et al. (1977). The apparatus consisted of a plexiglass cylinder (26.6 cm in height and 12 cm in diameter) filled with water (25°C) up to a height of 15 cm. Mice were placed individually into the cylinder, and their behavior was recorded by video camera for 10 min. Chlordiazepoxide, anxiolytic agent, did not affected the duration of immobility even in doses which produced noticeable ataxia, indicating this test does not reflect anxiety (Porsolt et al. 1978).

### Tail-suspension test

The tail-suspension test was performed according to the procedures described by Steru et al. (1985). Mice were suspended one at a time, 30 cm above the floor, by attaching an adhesive tape to the tip of the tail, and their behavior was recorded over a 5-min test period. Mice were considered to be immobile only when they hung passively and stayed motionless.

### Behavioral tests analysis

We used the analytical system for the behavioral studies developed by O'Hara & Co., based on movies recorded by an equipped video camera (2 frames/sec) in behavioral test apparatus and analysis software (Image LD, Image OF, Image EP, Image FZ), running on the public domain NIH Image and ImageJ programs (developed by Wayne Rasband at the National Institute of Mental Health). This analytical system enables us to acquire and analyze data of location, moving speed, moving distance, and immobility of mice automatically.

### Statistical analysis

Data were expressed as the mean ± SE. Comparisons among three groups were performed by one-way ANOVA, followed by a Tukey–Kramer multiple comparison test, when appropriate. A value of p < 0.05 was considered to be significant. Statistics were calculated with Excel-Toukei 2006 (Social Survey Research Information Co., Ltd.).

## Results

Table 2 shows the fatty acid composition of each dietary fat. Fish oil contained distinctive kinds of fatty acids, compared to the other fats. Noticeably, fish oil contained 20.2% EPA and 8.1% DHA, which were not detected in soybean oil or lard. Because of the high EPA and DHA concentration, the n-6/n-3 PUFA ratio was much lower in fish oil compared with soybean oil and lard. As is well known, fewer PUFA were included in lard than fish oil and soybean oil.

**Table 2:** **The fatty acid composition of the oils in experimental diets (%)**

	Fatty acid	Soybean oil	Lard	Fish oil
12:0	Lauric acid	-	2.826	0.070
14:0	myristic acid	5.551	2.583	8.133
15:0	pentadecylic acid	-	-	2.199
16:0	palmitic acid	5.661	25.073	15.937
16:1	palmitoleic acid	-	2.376	9.886
17:0	margaric acid	-	-	2.512
18:0	stearic acid	10.605	7.310	3.011
18:1	oleic acid	20.657	46.419	11.473
18:2n-6	linoleic acid	50.424	10.064	0.859
18:3n-3	α-linolenic acid	5.182	0.707	0.575
20:1	gadoleic acid	-	-	0.712
20:4n-6	arachidonic acid	-	0.384	0.959
20:5n-3	eicosapentaenoic acid	-	-	20.180
22:5n-3	docosapentaenoic acid	-	-	2.167
22:6n-3	docosahexaenoic acid	-	-	8.114
	other fatty acid	1.921	2.259	13.213
	n-6/n-3	9.7	14.8	0.1

**Figure 1:**
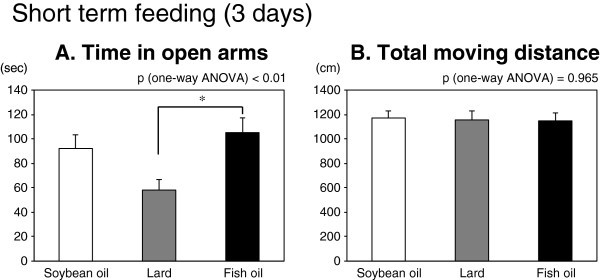
**The effect of short term feeding (3 days) of a particular diet containing different dietary fats (soybean oil, lard, or fish oil) on the anxiety-like behavior in the elevated plus-maze test.** Mouse behavior was tested for a 10-min interval in the elevated plus-maze. Increase of the time in open arms (**A**) is considered an anxiolytic index, whereas the total moving distance (**B**) is considered an index of general locomotor activity. Values are means ± SE for 24 mice per group. Significantly different between 2 groups (*p < 0.05, by Tukey-Kramer multiple comparison test). P-values by one-way ANOVA are shown above each graph.

In the 3-day short term feeding experiment, an obvious difference was observed in the elevated plus-maze test. The time spent on open arms was longest in the fish oil-fed group, followed by the soybean oil- and lard-fed group; there was a significant difference among three groups (Figure 1A, p < 0.01 by one-way ANOVA). The post hoc analysis showed there was a significant difference between the fish oil-fed group and the lard-fed group (p < 0.01 by Tukey-Kramer test); there was no significant difference between the soybean oil-fed group and fish oil-fed group, but there was a tendency (p = 0.077) between the soybean oil-fed group and lard-fed group. The locomotor activity in this test, shown as the total moving distance, was quite similar among three groups (Figure 1B); therefore, the difference of time spent in the open arms was not due to the difference of the locomotor activity. Meanwhile, testing with the other behavioral tests both assessing anxiety and depression did not show any significant differences among three groups (Table 3). There was also no difference among the three diet groups administered reserpine in the percentage immobility in the Porsolt forced-swim test and tail-suspension test. Depression was clearly induced by reserpine, indicated by the comparison between all mice injected vehicle versus reserpine: 29.6 ± 1.3 vs 39.0 ± 2.0% immobilization for the Porsolt forced-swim test and 24.5 ± 2.3 vs 57.2 ± 2.1% immobilization for the tail-suspension test (p < 0.01 by t-test, n = 72; 24 mice/group × 3 diet groups).

**Table 3:** **The behavioral test parameters of short term feeding experiment excluding elevatad plus-maze test**

Behavioral test	Parameters	Soybean oil	Lard	Fish oil
Light–dark transition test	Time in dark (s)	360.9 ± 13.1	370.8 ± 10.1	365.6 ± 8.5
	Time in light (s)	267.8 ± 12.7	257.8 ± 9.8	259.2 ± 8.1
	Number of transitions	47.6 ± 3.5	46.6 ± 2.4	47.7 ± 2.9
	Latency to enter light (s)	39.3 ± 5.9	50.1 ± 8.5	36.8 ± 8.2
Open field test	Total distance (cm)	4435.7 ± 160.3	4234.6 ± 149.3	4194.3 ± 128.4
	Time in central area (s)	77.9 ± 5.7	74.2 ± 4.4	76.2 ± 4.6
Social interaction test	Social area (s)	132.8 ± 7.2	139.2 ± 6.8	151.4 ± 6.8
	Nonsociar (s)	74.4 ± 6.5	73.3 ± 6.5	72.3 ± 6.3
	Other area (s)	392.8 ± 7.1	387.5 ± 7.9	376.4 ± 8.4
Porsolt forced-swim test	Immobility (%)	26.6 ± 2.1	29.4 ± 2.3	32.9 ± 2.5
Tail-suspension test	Immobility (%)	25.9 ± 5.0	23.1 ± 3.8	24.5 ± 3.3
Porsolt forced-swim test (reserpine-treated)	Immobility (%)	42.7 ± 3.6	35.8 ± 3.4	38.6 ± 3.3
Tail-suspension test (reserpine-treated)	Immobility (%)	57.4 ± 2.9	54.5 ± 3.9	59.6 ± 3.9

Values are means ± SE for 24 mice.

**Figure 2:**
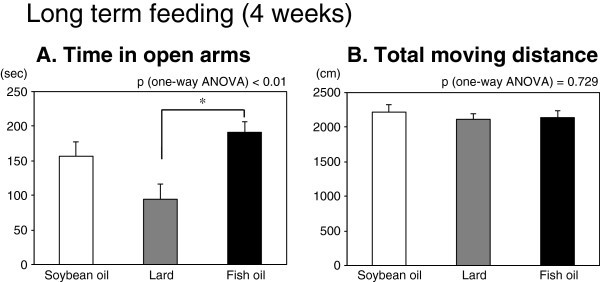
**The effect of long term feeding (4 weeks) of a particular diet containing different dietary fats (soybean oil, lard, or fish oil) on the anxiety-like behavior in the elevated plus-maze test.** Mouse behavior was tested for a 10-min interval in the elevated plus-maze. Increase of the time in open arms (**A**) is considered an anxiolytic index, whereas the total moving distance (**B**) is considered an index of general locomotor activity. Values are means ± SE for 12 mice per group. Significantly different between 2 groups (*p < 0.05, by Tukey-Kramer multiple comparison test). P-values by one-way ANOVA are shown above each graph.

In the 4-week long term feeding experiment, the time spent on open arms in the fish oil-fed group was significantly longer than that of the lard-fed group in the elevated plus-maze test (Figure 2A), similar to the result for the 3-day short term feeding experiment. There was also no significant difference in the locomotor activity in this test (Figure 2B). The other behavioral tests did not show any significant differences among the three groups (Table 4).

**Table 4:** **The behavioral test parameters of long term feeding experiment excluding elevated plus-maze test**

Behavioral test	Parameters	Soybean oil	Lard	Fish oil
Light–dark transition test	Time in dark (s)	354.4 ± 12.5	358.8 ± 15.3	352.1 ± 12.7
	Time in light (s)	252.0 ± 12.6	250.8 ± 15.1	255.2 ± 12.7
	Number of transitions	36.8 ± 2.7	36.9 ± 1.9	33.2 ± 2.7
	Latency to enter light (s)	44.9 ± 6.9	44.8 ± 6.1	41.0 ± 6.0
Open field test	Total distance (cm)	4098.3 ± 125.9	3851.4 ± 155.9	3638.9 ± 177.1
	Time in central area (s)	103.8 ± 13.4	92.2 ± 10.8	118.5 ± 12.1
Social interaction test	Social area (s)	162.9 ± 8.3	165.2 ± 7.3	183.8 ± 11.5
	Nonsociar (s)	78.9 ± 5.6	71.8 ± 4.6	72.1 ± 5.4
	Other area (s)	358.3 ± 9.0	363.0 ± 5.5	344.1 ± 8.4
Porsolt forced-swim test	Immobility (%)	18.5 ± 2.8	18.4 ± 1.5	18.9 ± 1.5
Tail-suspension test	Immobility (%)	30.0 ± 3.9	32.3 ± 5.0	21.4 ± 5.3
Porsolt forced-swim test (reserpine-treated)	Immobility (%)	29.8 ± 6.3	32.0 ± 5.9	35.7 ± 5.0
Tail-suspension test (reserpine-treated)	Immobility (%)	61.1 ± 7.3	69.3 ± 5.8	74.6 ± 4.7

Values are mean ± for 12 mice.

**Figure 3:**
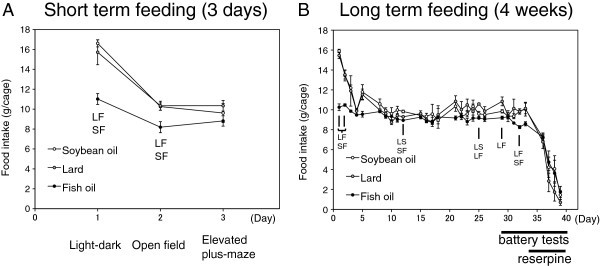
**The food intake of mice fed particular diet containing different dietary fat (soybean oil, lard or fish oil) in the short term feeding (3 days) (A) or long term feeding experiment (4 weeks) (B).** Values are means ± SE for 6 cages per group in (**A**) and 3 cages per group in (**B**). Each cage contained 4 mice. In the short term feeding experiment, the body weight and food intake of mice for the light–dark transition test, the open field test, and the elevated plus-maze test was shown. Two letters indicate a significant difference between two groups described by each initial: L is lard-fed, S is soybean oil-fed, and F is fish oil-fed group (p < 0.05, by Tukey-Kramer multiple comparison test and one-way ANOVA test).

The food intake during the experiment was shown in Figure 3. The food intake by the soybean oil-fed or the lard-fed group was transiently and significantly higher for the first 2 days than the fish oil-fed group in the short term feeding experiment; however, the food intake was not significantly different on the day of the elevated plus-maze test (Figure 3A). The food intake showed a similar trend in the first two days in the long term feeding experiment, but the food intake was close among the three groups during the rest of the experimental period (Figure 3B). Since the difference of food intake might affect results in the behavioral experiment, we compared the food restricted mice to the mice allowed ad libitum access to the commercial diet CRF-1 using the elevated plus-maze test, which simulated the proportion of food intake in the fish-oil fed group compared with the lard-fed group. As a result, there was no difference observed in the time in open arms and total moving distance between the ad libitum fed and food-restricted groups (Additional file 1: Figure S1), suggesting that the differences in behavior between the fish oil-fed and the lard-fed groups were not due to the difference of food intake.

## Discussion

The present study showed that mouse behavior was significantly less anxious when fed fish oil compared to lard in both relatively acute (3 days) and chronic (4 weeks) treatments. This was determined using the elevated plus-maze test, which uses the time spent on the open arms as an indicator of the level of anxiety. The group fed fish oil spent more time in the open arm area of the plus-maze than the group fed lard. Note that these results can also be interpreted as intake of lard increases anxiety-like behavior more than fish oil, depending on the perspective. Interestingly, the group fed soybean oil showed intermediate values between the fish oil-fed and lard-fed groups, and the differences between the soybean oil-fed and the other two groups were not statistically significant by post hoc ANOVA test.

In the fish oil-fed group, the increase of the time spent on the open arm in the elevated plus-maze test showed the same pattern in both the short term (within 3 days) and the long term (for 4 weeks) feeding. These results imply that the effect of fish oil on anxiety-like behavior of mice appears fairly rapidly, within 3 days from the start of dietary intake, and lasts for at least 4 weeks. However, such acute effects were hardly referred at present. In many reports, chronic administration of these diets for months was required before any improvement in anxiety symptoms was observed in either humans or rodents (Frances et al. 1995; Gamoh et al. 2001; Gamoh et al. 1999; Mills and Ward 1986; Nakashima et al. 1993; Watanabe et al. 2004; Yamamoto et al. 1988). Our finding clearly indicates there is a relatively acute effect of dietary fat on animal behavior.

Although the underlying alterations of the central nervous activity by the n-3 PUFA intake remains largely unknown, the relationship between n-3 PUFA deficiency and the endocannabinoid system is indicated. Lifelong n-3 PUFA deficiency induced significant anxiogenic behavior in the open field test, and ablates the long-term synaptic depression mediated by endocannabinoids in the prelimbic prefrontal cortex and accumbens that have been implicated in emotional behavior and mood disorders (Lafourcade et al. 2011). Moreover, in n-3 deficient mice, the effect of the cannabinoid agonist WIN55,212-2 in an anxiety-like behavior test was abolished and the cannabinoid receptor signaling pathways were altered in the prefrontal cortex and the hypothalamus (Larrieu et al. 2012). Therefore, the increase of the time spent on the open arm in the elevated plus-maze in the fish oil-fed group can link to an alteration in the endocannabinoid system in specific brain areas.

In contrast to anxiety-like behavior, the mice did not show behavioral differences for two other tests that evaluate depression, namely the Porsolt forced-swim test and tail-suspension test. This was the case in healthy mice as well as in mice with depression induced by reserpine. This finding suggests that dietary oils had little effect on the development of a depressed mood in mice and, interestingly, is not in accordance with previous results in humans, where intake of n-3 PUFA was shown to reduce symptoms of depression (Peet and Horrobin 2002). The self-reported symptom of depression in humans is much more complicated than what is measured by the two behavioral tests in mice; so, it is not surprising that these two results do not correspond. Furthermore, a recent report showed no association between the dietary intake of n-3 PUFA or fish consumption and depression, major depressive episodes, or suicide (Hakkarainen et al. 2004). Therefore, the relation between fish oil or n-3 PUFA intake and depression can best be described at present as inconclusive.

The elevated plus-maze test, light–dark transition test, open field test, and social interaction test are used to assess anxiolytic behaviors in mice. In this experiment, only the elevated plus-maze test showed a difference with n-3 PUFA intake. In the behavioral battery tests, even the same mutant mice display different results in tests to assess anxiety (Holmes et al. 2003; Miyakawa et al. 2003). Probably, each test must reflect various and different aspects of anxiety, and the extent of fear to induce anxiety must not be same. A principal component analysis revealed that the variables from the elevated plus-maze were highly correlated with the locomotion and exploration factor, whereas the light–dark transition test was partly related to neophobia factor (Belzung and Le Pape 1994). The elevated plus-maze is known to increase the concentrations of plasma stress hormones (File et al. 1994; Rodgers et al. 1999). A significantly greater ACTH response was revealed after the elevated plus-maze exposure compared to the exposure to the light–dark transition test in C57BL/6J mice (Holmes et al. 2003), suggesting the extent of fear can be different in each test. It is also reported that the light–dark transition test showed to be less sensitive to anxiolytic drugs than the elevated plus-maze test (Chaouloff et al. 1997). This issue was pointed in a previous report by Ramos et al. (Ramos et al. 2008), as data from correlational studies, in spite of being somewhat contradictory (Ramos et al. 1997; Ramos et al. 1998), often indicate that there is little correlation among anxiety-related behaviors measured in different tests (File 1991; Ramos et al. 1998; Trullas and Skolnick 1993). Alternatively, it was possible that the effect of dietary oil emerged only from Day 3, when the light–dark transition test and open field test had already finished in the short term feeding experiment. However, if it occurred, we must have found some differences in the light–dark transition test and open field test in the long term feeding experiment.

The elevated plus-maze test is currently the first-choice test for screening anxiolytic drugs. Pharmacological treatments showed that two benzodiazepine anxiolytics and one anxiogenic respectively increased and decreased the approach to the open arms. Treatments with seven antidepressants with different pharmacological profiles had no effect on arm preferences (Ramos and Mormede 1998). These results indicated that our results observed in the elevated plus-maze test were associated with anxiety but not depression.

## Conclusions

Dietary intake of fish oil in this study was found to attenuate anxiety-like behavior in mice better than intake of other dietary fats, such as lard. We were surprised to observe this effect after only 3 days of dietary intake, as well as an effect after 4 weeks of intake. These findings suggest that dietary fat type does influence the behavioral signs of anxiety fairly rapidly.

## Authors’ information

Present address of Yusuke Sato: Department of Animal Science, Utsunomiya University, Utsunomiya, Japan.

## Electronic supplementary material

Additional file 1: Figure S1: The effect of food restriction on the anxiety-like behavior in the elevated plus-maze test. Mouse behavior was tested for a 10 minute interval in the elevated plus-maze. Increase of the time in open arms (**A**) is considered an anxiolytic index, whereas the total moving distance (**B**) is considered an index of general locomotor activity. Nine-week-old male C57BL/6J mice (n = 8/group) were given free access to water and a diet for 1 week prior to the start of the experiments. Then the food-restricted group was given 67%, 85% and 88% of food intake on Day 1, 2 and 3, respectively. The elevated plus-maze test was done on Day 3. As a result, there was no difference observed in the result of time in open arms and total moving distance between the ad libitum fed and food-restricted group, suggesting that the differences in behavior between the fish oil-fed and the lard-fed groups were not due to dietary restriction. On the contrary, it is reported that a 50% food restriction for 5 weeks caused a significant decrease (rather than an increase) of the time spent on open arms during the elevated plus-maze test in rats (Jahng et al. 2007). P-values by unpaired *t*-test are shown above each graph. (PDF 21 KB)

## Abbreviations

PUFA: Polyunsaturated fatty acid
DHA: Docosahexaenoic acid
EPA: Eicosapentaenoic acid.
