# Supplementary material for: Effect of dietary fat type on anxiety-like and depression-like behavior in mice
Source: Springerplus. 2013 Apr 16;2:165. doi: 10.1186/2193-1801-2-165 (PMC3647083; doi:10.1186/2193-1801-2-165)
Supplement: Supplementary file 1 — Additional file 1: Figure S1: The effect of food restriction on the anxiety-like behavior in the elevated plus-maze test. Mouse behavior was tested for a 10 minute interval in the elevated plus-maze. Increase of the time in open arms (A) is considered an anxiolytic index, whereas the total moving distance (B) is considered an index of general locomotor activity. Nine-week-old male C57BL/6J mice (n = 8/group) were given free access to water and a diet for 1 week prior to the start of the experiments. Then the food-restricted group was given 67%, 85% and 88% of food intake on Day 1, 2 and 3, respectively. The elevated plus-maze test was done on Day 3. As a result, there was no difference observed in the result of time in open arms and total moving distance between the ad libitum fed and food-restricted group, suggesting that the differences in behavior between the fish oil-fed and the lard-fed groups were not due to dietary restriction. On the contrary, it is reported that a 50% food restriction for 5 weeks caused a significant decrease (rather than an increase) of the time spent on open arms during the elevated plus-maze test in rats (Jahng et al. 2007). P-values by unpaired t-test are shown above each graph. (PDF 21 KB) [file 40064_2012_235_MOESM1_ESM.pdf]

Supplemental Data

Figure S1 The effect of food restriction on the anxiety-like behavior in the elevated plus-maze test. Mouse behavior was tested for a 10 minute interval in the elevated plus-maze. Increase of the time in open arms (A) is considered an anxiolytic index, whereas the total moving distance (B) is considered an index of general locomotor activity. Nine-week-old male C57BL/6J mice (n = 8/group) were given free access to water and a diet for 1 week prior to the start of the experiments. Then the food-restricted group was given 67%, 85% and 88% of food intake on Day 1, 2 and 3, respectively. The elevated plus-maze test was done on Day 3. As a result, there was no difference observed in the result of time in open arms and total moving distance between the ad libitum fed and food-restricted group, suggesting that the differences in behavior between the fish oil-fed and the lard-fed groups were not due to dietary restriction. On the contrary, it is reported that a 50% food restriction for 5 weeks caused a significant decrease (rather than an increase) of the time spent on open arms during the elevated plus-maze test in rats (Jahng et al. 2007). P-values by unpaired *t*-test are shown above each graph.

Supplemental References

Jahng JW, Kim JG, Kim HJ, Kim BT, Kang DW, Lee JH. (2007) Chronic food restriction in young rats results in depression- and anxiety-like behaviors with decreased expression of serotonin reuptake transporter. Brain Res 1150: 100-107

Fig. S1

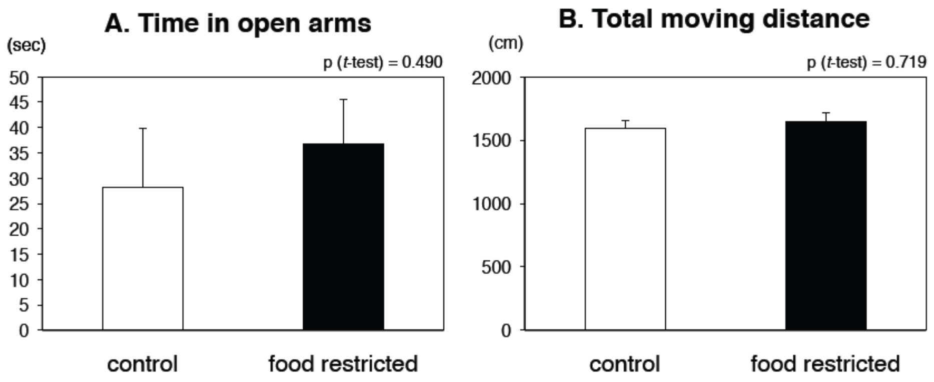

Mizunoya et al.
